# Physiological changes in *Rhodococcus ruber* S103 immobilized on biobooms using low-cost media enhance stress tolerance and crude oil-degrading activity

**DOI:** 10.1038/s41598-022-14488-0

**Published:** 2022-06-21

**Authors:** Kallayanee Naloka, Jirakit Jaroonrunganan, Naphatsakorn Woratecha, Nichakorn Khondee, Hideaki Nojiri, Onruthai Pinyakong

**Affiliations:** 1 Center of Excellence in Microbial Technology for Marine Pollution Treatment (MiTMaPT), Department of Microbiology, Faculty of Science, Chulalongkorn University, Bangkok, 10330 Thailand; 2 Research Program on Remediation Technologies for Petroleum Contamination, Center of Excellence on Hazardous Substance Management (HSM), Chulalongkorn University, Bangkok, 10330 Thailand; 3 Department of Natural Resources and Environment, Faculty of Agriculture, Natural Resources and Environment, Naresuan University, Phitsanulok, 65000 Thailand; 4 Agro-Biotechnology Research Center, Graduate School of Agricultural and Life Sciences, The University of Tokyo, Tokyo, 113-8657 Japan; 5 Collaborative Research Institute for Innovative Microbiology, The University of Tokyo, Bunkyo-ku, Tokyo, 113-8657 Japan

**Keywords:** Biotechnology, Microbiology, Environmental sciences

## Abstract

For economic feasibility, sugarcane molasses (0.5%, w/v) containing K_2_HPO_4_ (0.26%, w/v) and mature coconut water, low value byproducts, were used in cultivation of *Rhodococcus ruber* S103 for inoculum production and immobilization, respectively. Physiological changes of S103 grown in low-cost media, including cell hydrophobicity, saturated/unsaturated ratio of cellular fatty acids and biofilm formation activity, enhanced stress tolerance and crude oil biodegradation in freshwater and even under high salinity (5%, w/v). Biobooms comprised of S103 immobilized on polyurethane foam (PUF) was achieved with high biomass content (10^10^ colony-forming units g^−1^ PUF) via a scale-up process in a 5-L modified fluidized-bed bioreactor within 3 days. In a 500-L mesocosm, natural freshwater was spiked with crude oil (72 g or 667 mg g^−1^ dry biobooms), and a simulated wave was applied. Biobooms could remove 100% of crude oil within only 3 days and simultaneously biodegraded 60% of the adsorbed oil after 7 days when compared to boom control with indigenous bacteria. In addition, biobooms had a long shelf-life (at least 100 days) with high biodegradation activity (85.2 ± 2.3%) after storage in 10% (w/v) skimmed milk at room temperature. This study demonstrates that the low-cost production of biobooms has potential for future commercial bioremediation.

## Introduction

Crude oil spills are one of the most severe pollutants in aquatic ecosystems due to their toxicity and recalcitrance, causing short- and long-term effects on living organisms and human health at contaminated sites^1–3^. Several strategies have been established to clean up harmful hazardous materials from oil-polluted marine shorelines and freshwater ecosystems. However, although physical (skimmers, sorbent booms and floating barriers etc.) and chemical (dispersants, solidifiers and demulsifiers etc.) strategies can be used as initial management, they have some limitations, such as incomplete oil removal and the generation of secondary pollutants^4^. Therefore, bioremediation should be supplemented to achieve minimal impact on the environment^5,6^. Thus, two or more methods such as physical process and bioremediation should be combined to construct oil boom bioproduct that tend to be practical for completely cleaning up oil spills in aquatic environments^7^. In this study, biobooms refer to effective hydrocarbon-degrading bacteria immobilized on oil sorbent boom, that can remove oil from water and simultaneously biodegrade the adsorbed oils.

*Rhodococcus* species are highly effective in hydrocarbon bioremediation due to their strong adaptability to environmental stressors and diverse metabolic activities^8^. Among the genus members, *R. ruber* is one of the promising candidates for environmental bioremediation since it is a nonpathogenic species and ability to degrade a wide range of hydrocarbon compounds such as alkanes^9^, naphthalene^10^ and crude oil^11^. In our previous study, *R. ruber* S103 showed good performance in the degradation of various hydrocarbon compounds, high cell hydrophobicity, biosurfactant production and biofilm formation^12^. However, the success of bioaugmentation is strongly dependent on the survival and activity of exogenously inoculated microorganisms which may be affected by various abiotic factors^13^. Bacterial immobilization on carriers is a good method for decreasing the ratio of cell washout and crude oil-degrading activity^14–16^. Polyurethane foams (PUFs) have been used in environmentally oriented applications due to their relatively cheap commercial price per 1 kg of 2.5 to 4.5 US$^17–19^. PUFs are chemically crosslinked structures synthesized from polyisocyanates or polyols, making them lightweight materials with high surface areas for cell attachment and biofilm development and capable of decomposing in environments^19^. PUFs and particle-coated PUFs have good characteristics of high absorption ability of oils from water^20–22^. Additionally, PUFs could be biodegraded by microorganisms (bacteria, fungi and algae) and some new types was produced from biobased materials, making them a good carrier to reduce the detrimental environmental impacts^19^. Many reports on the use of PUF-immobilized bacteria for the removal of crude oil and its derivatives are available^23–25^. However, to be economically interesting, industrial production of immobilized cells requires low-cost culture media and sustainable bioprocesses^13,26^.

Using agroindustrial wastes such as sugarcane molasses and mature coconut water, inexpensive locally available materials in many countries, for bacterial biomass production should reduce costs^27–31^. To our knowledge, a few studies used molasses and coconut water as alternative carbon sources in a minimal medium for *Rhodococcus* species in bioprocesses^27–30^. There is no report to directly use as a medium for inoculum preparation and immobilization in bioremediation. Additionally, the carbon source had a great influence on changes in bacterial physiology, including cell hydrophobicity, fatty acid profiles and biofilm formation^32–34^. Previous studies have focused on changes in bacterial properties in response to stress after exposure to toxic hydrocarbons^33,35,36^, but the preparation of toxic tolerant cells for enhanced biodegradation activity has not been reported. Moreover, types of bioreactors have gained considerable importance for large-scale production^37^. A fluidized-bed bioreactor (FBB) has many advantages related to high-yield biomass, high treatment efficiency, less clogging and relatively low operating costs due to the low energy and small area required^38^. The FBB is one of the most common bioreactors to be used for wastewater biotreatment and microbial degradation of toxic pollutants^39,40^, but a few reports discuss direct use for bacterial immobilization^41^.

The main aims of this work were therefore to produce a ready-to-use bioproduct using a hybrid strategy of physical and biological treatments and investigate the feasibility of the bioremediation of crude oil contamination in real environments. Low-cost substrates were selected for inoculum preparation and immobilization of *R. ruber* S103. The physiological changes of S103, including cell hydrophobicity, cellular fatty acid and biofilm formation, resulting in toxic tolerance and crude oil-degrading activity under different salinities were evaluated. Scaling-up production of the PUF-immobilized S103 as biobooms was performed in a modified FBB. The biobooms were further applied to bioremediation of crude oil contaminated on a mesocosm scale as the simulated freshwater environment under natural conditions.

## Materials and methods

### Medium composition, low-cost substrate and crude oil

Luria–Bertani (LB) agar^42^ and 4-fold-diluted LB broth (0.25xLB) were used for viable cell counts and inoculum preparation, respectively. Molasses was purchased from the Mitr Phol Sugar Corporation Limited (Bangkok, Thailand). A molasses-based medium (0.5%MK) contained 0.5% (w/v) molasses and 0.26% (w/v) K_2_HPO_4_ to obtain pH 7.0. Mature coconut water (100%CW) was purchased from a coconut peeling factory in Samut Songkhram Province, Thailand. The 100%CW was adjusted to pH 7.0 with 1 M NaOH. The Arabian light (AL) crude oil source and properties are shown in Text S1.

### Evaluation of molasses and coconut water as alternative low-cost media

#### Inoculum preparation

A preculture of *R. ruber* S103 was grown in 250-mL Erlenmeyer flasks containing 100 mL of three assayed media, 0.25xLB, 0.5%MK and 100%CW. Flasks were incubated at room temperature (RT), 30–33 °C on an orbital shaker at 200 rpm for 24 h. The preculture was centrifuged to harvest cells at 8000 rpm and 4 °C for 10 min and washed twice with 0.85% (w/v) NaCl solution. Then, the cells were resuspended in the same solution to obtain the inoculum with 10^8^ CFU mL^−1^ viable cells (OD_600_ equal to 1.2). The cell suspension was further agitated overnight to allow the cells to utilize the accumulated nutrients before being used as an inoculum for hydrocarbon biodegradation.

#### Biomass production measurement

The inoculum (1 mL) was inoculated to obtain the initial cells of 10^6^ CFU mL^−1^ in 250 mL Erlenmeyer flasks containing 100 mL of 0.25xLB, 0.5%MK and 100%CW media. The flasks were incubated at RT, with shaking 200 rpm for 120 h. Bacterial growth was determined by viable cell counts on LB agar at RT for 3 days. The kinetics of bacterial growth were determined by sampling at specific time intervals. The specific growth rate was estimated visually as the exponential plot of bacterial growth versus incubation time and calculated by the equation; µ (h^−1^) = ln 2/td, where td is the doubling time for cell division^43^.

#### Biodegradation of crude oil by free-living S103 cells grown in alternative media

The inoculum (5 mL) with 10^8^ CFU mL^−1^ cells was added to 125-mL Erlenmeyer flasks containing 45 mL of carbon-free mineral medium (CFMM)^44^ supplemented with 2500 mg L^−1^ AL crude oil. Flasks were incubated at RT and 200 rpm for 60 h. Uninoculated medium with crude oil was used as a control. Bacterial growth was determined by viable cell counts on LB agar at RT for 3 days. Residual crude oil was extracted twice with 5 mL of chloroform. The extracted oil was evaporated to remove chloroform and then dissolved in 5 mL of *n*-hexane. The aliphatic compounds of AL crude oil was quantified using gas chromatography with a flame ionization detector (GC-FID; Agilent Technologies, USA) according to Nopcharoenkul et al.^45^.

#### Toxicity test of crude oil

Each inoculum (0.5 mL) with a cell concentration of 10^8^ CFU mL^−1^ was inoculated into 4.5 mL of CFMM containing various concentrations (2000–20,000 mg L^−1^) of AL crude oil. Cells were directly exposed to toxic crude oil for 12 h at RT and 200 rpm. Then, the remaining cells were enumerated by serial dilution on LB agar incubated at RT for 3 days. The survival (%) was calculated by comparison between numbers of the inoculum and cell after oil exposure.

#### Analyses of cell hydrophobicity and fatty acid profile

The inoculum (10 mL) was transferred to a 250-mL Erlenmeyer flask containing 90 mL of the different culture media (0.25xLB, 0.5%MK and 100%CW) and incubated at RT and 200 rpm for 2 days. The cultured cells (5 mL) were centrifuged at 8000 rpm and 4 °C for 10 min. Cell pellets were washed twice and suspended in phosphate urea magnesium sulfate (PUM) buffer^46^ or subjected to a cell hydrophobicity test according to Naloka et al.^12^. The remaining cultured cells were harvested by centrifugation and washed three times with sterile distilled water. Cell pellets were lyophilized using a freeze dryer (Eyela, Japan). The cellular fatty acids were analyzed by the Thailand Institute of Scientific and Technological Research, as instructed for the microbial identification system with MIDI software (Sherlock rapid method version 5.0).

#### Biofilm formation analysis

Biofilm formation was examined using a slightly modified method from Naloka et al.^12^. Briefly, the centrifuged cells of the preculture were diluted with each tested medium to obtain an initial cell concentration of 10^7^ CFU mL^−1^. The cell suspension (120 µL) was transferred into 96-well plates and statically incubated at RT for 5 days. After incubation, the biofilm coated on the plate was stained with crystal violet and destained with ethanol. The crystal violet-ethanol solution was transferred into a new plate and quantified at 540 nm using a multimode plate reader (PerkinElmer, Finland). The wells containing the uninoculated media were used as blank controls.

#### Immobilization of S103 on PUF

PUF cubes (1 × 1 × 1 cm^3^) with a density of 18.6 g L^−1^ were purchased from the Thai EPP Foam Company Limited (Thailand). They were used directly without any pretreatment before immobilization to reduce the production costs. PUF (0.3 g dry weight) was soaked in 90 mL of each medium (0.25xLB, 0.5%MK and 100%CW) contained in 250-mL Erlenmeyer flasks before sterilization and inoculated with 10 mL of inoculum (10^8^ CFU mL^−1^). Flasks were incubated at RT and 200 rpm. The immobilized cells were enumerated daily for 5 days as described by Naloka et al.^12^. The morphology of the immobilized cells was visualized by scanning electron microscopy (SEM) using a JSM-IT500HR model (JEOL Ltd., Tokyo, Japan) according to Sakdapetsiri et al.^47^.

### Scale-up production of biobooms in a modified FBB

S103 was precultured in a 250-mL Erlenmeyer flask containing 130 mL of 0.5%MK at RT and 200 rpm for 1 day. The 10% (v/v) inoculum (10^8^ CFU mL^−1^) was transferred into 2 L of FBB containing 0.3% (w/v) PUF soaked in 1.35 L of 100%CW. The operation was performed with filtered air at 1.50 L min^−1^ at RT for 3 days. The samples were taken daily to measure the bacterial number of immobilized cells. Moreover, crude oil removal in a simulated environment, the scale-up production of biobooms was carried out in a modified FBB as a 5-L Erlenmeyer flask containing 0.3% (w/v) PUF soaked in 2.6 L of 100%CW and 10% (v/v) inoculum. The operation was performed at RT with filtered air at 2.86 L min^−1^ for 3 days.

### Validation of efficient biobooms for crude oil removal at the flask scale

The efficient removal of AL crude oil by biobooms was assessed in CFMM. Biobooms (0.3 g dry weight) with 10^9^ CFU g^−1^ PUF were rested into 250-mL Erlenmeyer flasks containing 100 mL of sterilized 0.85% NaCl for 24 h and then transferred into 100 mL of CFMM supplemented with 2500 mg L^−1^ AL crude oil (667 mg g^−1^ dry boom or bioboom) and incubated at RT and 200 rpm for 3 days. The abiotic controls were CFMM containing crude oil with and without the sterilized PUF. The samples were collected daily to analyze residual crude oil. The cell survival of the immobilized S103 was estimated by viable cell count on LB agar.

Crude oil removal of biobooms was further examined in freshwater supplemented with 2500 mg L^−1^ AL crude oil (667 mg g^−1^ dry boom or bioboom). Freshwater was collected from the Chao Phraya River, and the physicochemical properties are shown in the Supplementary Table S1. The crude oil removal efficiency was determined as described above. Three controls were sterilized freshwater containing crude oil with or without sterilized PUF and nonsterilized freshwater with crude oil (natural attenuation). The samples were taken at 0, 1, 3, 5 and 7 days of incubation to analyze residual oil and survival of S103.

### Efficiency assessment of biobooms on crude oil removal in simulated environmental contamination

The experimental procedure was conducted in circular plastic tanks, sized 110 cm × 80 cm (diameter × height), as shown in the Supplementary Fig. S1a. All tanks contained 72 g of AL crude oil in 500 L of nonfiltered and nonsterilized freshwater pumped directly from the Tha Khoei Canal, Ratchaburi Province (13°26′26.5"N 99°23′39.8"E), with physicochemical properties listed in the Supplementary Table S1. In the 1st tank (natural attenuation), no special treatment was performed apart from applying simulated wave frequency (50 Hertz and 36 Watt) using the aquarium power heads model (Crab Aqua Technology, China). The 2nd tank (control) contained an oil sorbent boom as an uninoculated PUF, while the 3rd tank (bioaugmentation) was remediated with biobooms. The 108 g (dry weight) of uninoculated PUF and bioboom was packed in nylon net and then roped on the middle of mesocosm tanks (Supplementary Fig. S1b). Both mesocosms (tanks 2 and 3) were supplemented with 72 g of crude oil to obtain a final concentration of 667 mg g^−1^ dry booms or biobooms which was equal to the flask scale. In the sampling strategy, 50 mL of water was aseptically taken at 4 points and 3 depths (surface, 30- and 60-cm depth) using a sterile conical tube and silicon tube. The 24 pieces of PUF cubes (equal to 0.3 g dry weight) were collected from different area of booms and biobooms treatments in triplicate (Supplementary Fig. S1b). The samples were taken at specific intervals (0, 3 and 7 days) to analyze residual crude oil and survival of S103.

### Evaluation of storage stability of bioboom product

The biobooms were washed twice with 50 mL of phosphate buffer (pH 6.5). Then, 0.3 g dry weight of biobooms (S103 cells of 10^9^ CFU g^−1^ PUF) was added to a 50-mL sterile conical tube, and 40 mL of preservative buffer, phosphate buffer and 10% (w/v) skimmed milk, were added to fill the space within the tube. The storage conditions were varied at 4 °C and RT for 30, 60 and 100 days. The samples were analyzed for survival of S103 and crude oil removal.

### Statistical analysis

Experiments were conducted in triplicate, and the values obtained were reported as the mean ± standard deviation of triplicates. Analysis of variance (ANOVA) was used for statistical analysis using SPSS software version 22.0. Duncan’s test for multiple comparisons was applied to express significant differences between the means at the 95% confidence level (*P* < 0.05).

## Results and discussion

### Molasses and coconut water as alternative low-cost media

#### Biomass production

Regarding cost-effective production for industrial applications, low-cost media with different concentrations were preliminarily screened for the growth of *R. ruber* S103 (Text S2). A typical growth of S103 was performed to ensure that the selected medium composition could increase biomass production. At 24 h for cultivation, S103 showed better growth in alternative media, 100%CW and 0.5%MK, than chemically synthetic 0.25xLB medium (Supplementary Fig. S5). The specific growth rates of S103 grown in these media were calculated from 0 to 24 h (Table 1). This is clear that rapid growth of S103 was significantly promoted in the alternative media, 100%CW and 0.5%MK, because both media are rich in carbon, nitrogen, organic acids, vitamins and trace elements (Supplementary Table S2). Additionally, CW and molasses contained higher carbon-to-nitrogen (C/N) ratios, when compared to LB medium analyzed by Mouginot et al.^48^ (Table 1). Generally, CW and molasses are used as alternative carbon sources in mineral salt media for *Rhodococcus* species in many biotechnological aspects but not biomass production for crude oil biodegradation^27–30^. This study successfully used inexpensive organic substrates locally available in food processing wastes without adding any nutrients except an adequate inorganic buffer for enhanced biomass production of *R. ruber* S103.

**Table 1:** Specific growth rate of *R. ruber* S103 grown in different culture media and their carbon to nitrogen ratio.

Culture medium	Specific grow rate (h^−1^)	Carbon to nitrogen ratio (C/N)
100%CW	0.4	16.8
0.5%MK	0.3	6.1
0.25xLB	0.2	3.9*

*C/N ratio of LB was obtained from Mouginot et al.^48^.

#### Biodegradation of crude oil by free-living S103 cells grown in alternative media

**Figure 1:**
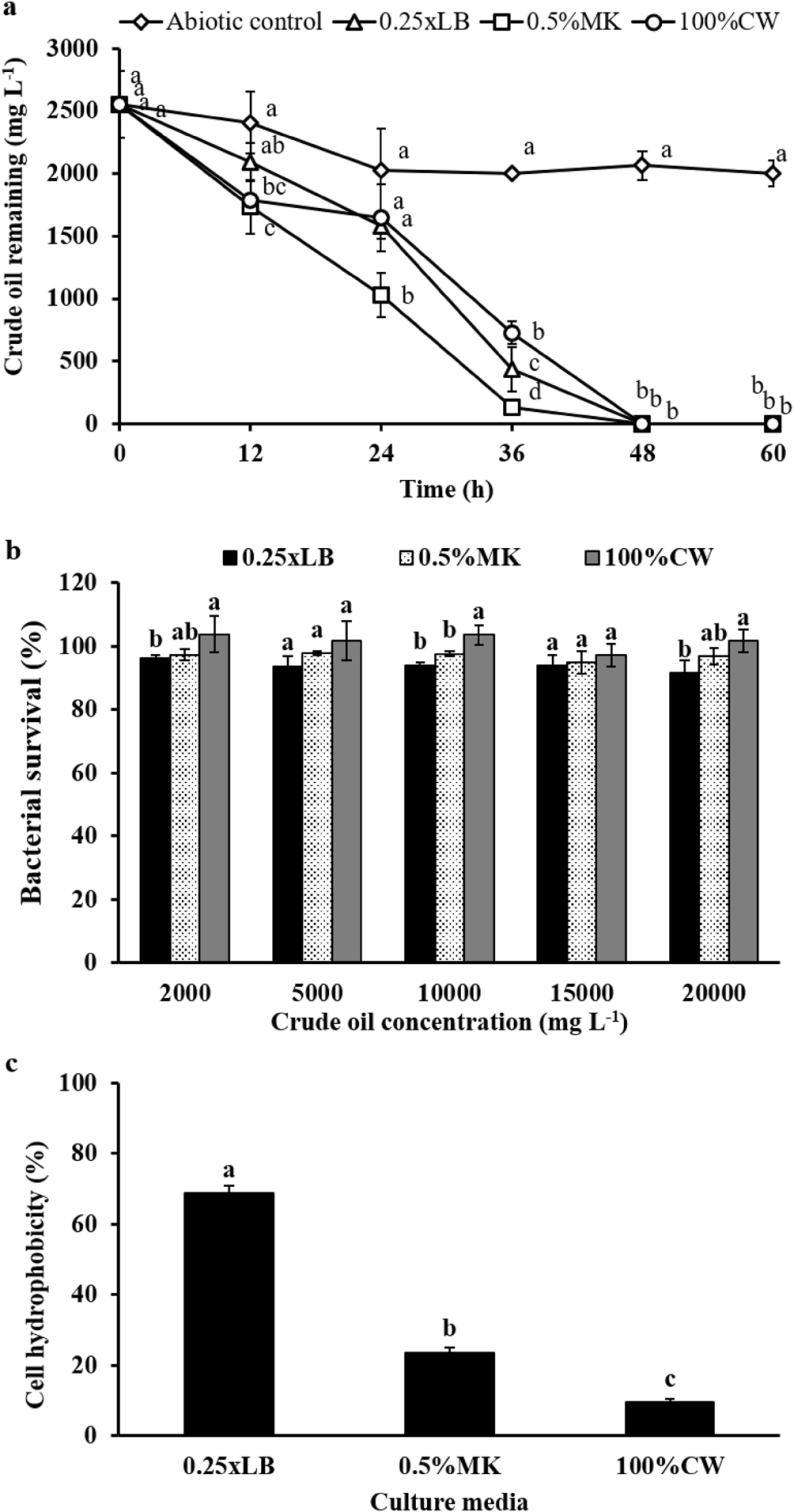
Biodegradation efficiency of *R. ruber* S103 in CFMM medium supplemented 2500 mg L^−1^ AL crude oil at room temperature (30–33 °C) and 200 rpm for 60 h (**a**). Bacterial survival of S103 after exposure to crude oil in CFMM medium for 12 h (**b**). Cell hydrophobicity of S103 grown in different media, 0.25xLB, 0.5%MK and 100%CW (**c**). Preculture of S103 was separately prepared in three media. The lowercase letters above the vertical bars represent significant differences (*P* < 0.05) of the same day (**a**), each concentration (**b**) and medium (**c**).

*Rhodococcus ruber* S103 is a promising hydrocarbon degrader for the practical bioremediation of crude oil contamination since it effectively degraded 99.8% of 2000 mg L^−1^ AL crude oil and more than 85% of crude oil at concentrations up to10,000 mg L^−1^ (Supplementary Fig. S6a), with cell survival of approximately 10^6^ CFU mL^−1^ in liquid CFMM after 7 days (Supplementary Fig. S6b). S103 precultured in 0.25xLB, 0.5%MK and 100%CW was further compared for the biodegradation of 2500 mg L^−1^ crude oil in CFMM. S103 was found to effectively degrade crude oil to below the detection limit within 48 h even if it was precultured in alternative media. Meanwhile, S103 grown in 0.5%MK exhibited faster crude oil biodegradation than other precultured cells (Fig. 1a). Interestingly, at 12 h of cultivation, S103 grown in 0.5%MK and 100%CW showed higher crude oil degradation than cells grown in 0.25xLB (Fig. 1a). Furthermore, the toxicity test after direct exposure to various crude oil concentrations (2000–20,000 mg L^−1^) for 12 h confirmed that the survival of S103 tended to increase when the cells were precultured in 0.5%MK and 100%CW, compared to 0.25xLB (Fig. 1b), which might be related to changes in some physiological properties.

#### Changes in S103 cell physiological properties induced by alternative media

The hydrophobicity of S103 cells grown in different media was determined. The results showed that cell hydrophobicity was significantly decreased by S103 grown in both media compared with 0.25xLB (Fig. 1c). This might be that both media rich in various sugars as a carbon source (Supplementary Table S2) resulted in alteration of bacterial cell wall structure. It is well-known that physiological microorganisms (cell wall structure, virulence, biofilm formation, or antibiotic resistance) are relevant on carbon sources for growth such reports in *Pseudomonas aeruginosa* PA14^49^ and in *Candida albicans*^50^. The proteomic analysis should be evaluated to describe more details in future. Moreover, high cell hydrophobicity of S103 from 0.25xLB might increase the rapidly facilitated diffusion of crude oil after immediate exposure through physical interaction with microbial cells, resulting in growth inhibition of S103. Bacteria with hydrophobic cell walls have a higher affinity for hydrophobic compounds than bacteria with more-hydrophilic cell walls^51^. Kaczorek et al.^32^ reported that the cell hydrophobicity of bacteria is correlated with carbon sources in the culture medium and that lipophilic hydrocarbons could have toxic effects through the interaction of these compounds with membrane constituents. Cell hydrophobic properties seem to be the most important factor in hydrocarbon utilization; however, Obuekwe et al.^52^ indicated that *Williamsia muralis* lacked demonstrable hydrophobic character but was still able to degrade crude oil (>53%), suggesting the existence of other mechanisms correlated with the physical contact between bacteria and petroleum hydrocarbons^53^.

Additionally, analysis of cellular-derivative fatty acids showed that S103 grown in low-cost media could be tolerant to toxic compounds because of an almost 2-fold higher saturated/unsaturated ratio than cells grown in 0.25xLB (Table 2). This phenomenon may be observed because saturated fatty acids show a higher degree of membrane ordering, which allows an increase in membrane rigidity. These effects are known to oppose the partition of hydrophobic compounds to a lipid bilayer, contributing to stress tolerance^51,54^. For instance, *Rhodococcus aetherivorans* BCP1 changes in cell morphology and fatty acid composition under naphthenic acids enhances stress resistance^55^. Therefore, these results suggest that the use of low-cost media for biomass production not only improves the strong cell wall to resist toxicity, but also promotes crude oil biodegradation. After careful comparison with regard to nutrient costs and biodegradation, 0.5%MK was selected as an alternative medium for inoculum preparation of S103.

**Table 2:** Cellular fatty acid composition of *R. ruber* S103 cultivated in different media.

Fatty acid	Content (% dry weight)		
	0.25xLB	0.5%MK	100%CW
**Saturated**			
C10:0	–	–	0.64
C10:0 iso	–	–	0.65
C12:0	0.11	0.16	0.39
C13:0	0.05	0.08	–
C14:0	3.41	3.90	4.53
C15:0 anteiso	0.06	0.04	0.09
C16:0	31.14	39.23	43.96
C16:0 iso	0.06	0.12	–
C16:0 3-OH	–	–	0.08
C16:0 10-methyl/C17:1 iso *ω*9c*	0.97	1.80	–
C16:0 N alcohol	–	–	0.57
C17:0	2.89	1.84	1.68
C17:0 anteiso	0.07	0.26	0.14
C17:0 iso	–	0.16	–
C17:0 iso 3-OH	–	–	0.38
C17:0 10-methyl	0.84	0.69	0.12
C18:0	1.21	2.42	10.35
C18:0 3-OH	0.07	–	0.32
C18:0 10-methyl	11.76	13.54	1.75
C19:0	–	–	0.18
C20:0	1.03	0.54	0.82
C20:0 iso	0.09	–	–
Total	53.76	64.78	66.65
**Unsaturated**			
C15:1 anteiso A	–	–	0.31
C15:1 *ω*5*c*	0.32	0.13	–
C15:1 *ω*8*c*	0.15	0.20	–
C16:1 *ω*7*c* alcohol	–	–	0.63
C17:1 *ω*8*c*	2.71	1.62	0.56
C17:1 iso *ω*5*c*	–	–	0.48
C18:1 *ω*9*c*	17.52	14.70	24.22
C20:1 ω9c	–	–	0.26
C16:1 *ω*7*c*/*ω*6*c*	22.96	16.77	6.73
C19:1 *ω*11*c*/*ω*9c*	2.30	1.48	–
C19:1 *ω*7*c*/*ω*6 *c* *	0.29	0.17	–
C17:1 anteiso *ω*9*c*	–	0.15	0.15
Total	46.25	35.22	33.34
**Sat./Unsat. ratio**	**1.16**	**1.84**	**2.00**

*Represents groups of two fatty acids that could not be separated using the MIDI system.

OH: position of hydroxyl group from the acid end; *ω*: methyl end of fatty acid;

iso/anteiso: branched fatty acids; *c*: cis configuration of the double bound.

#### Biofilm formation and immobilization of S103 on PUFs

**Figure 2:**
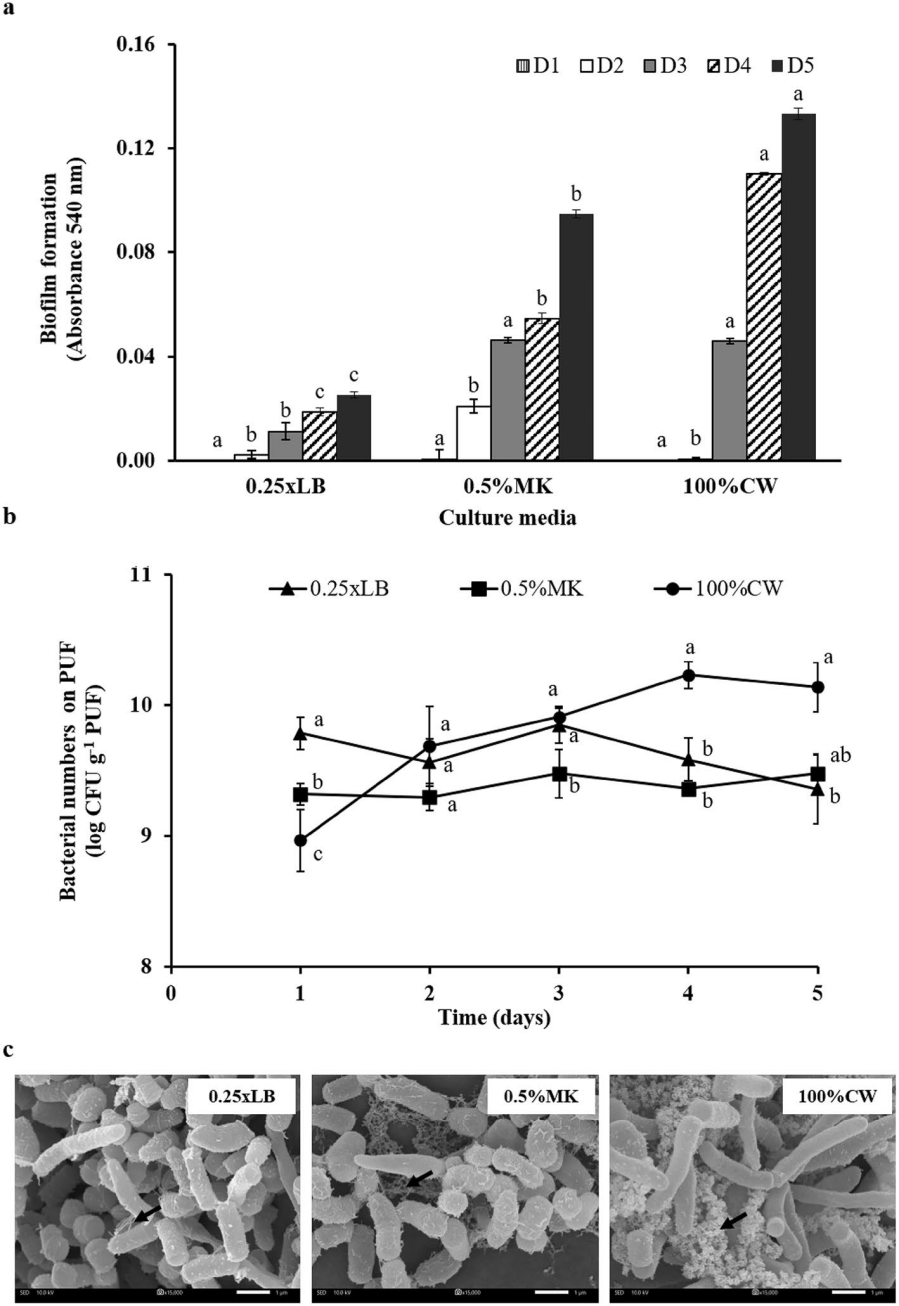
Biofilm formation of *R. ruber* S103 cultured in 0.25xLB, 0.5%MK and 100%CW and statically incubated at room temperature for 5 days (**a**). Bacterial numbers of S103 immobilized on PUF in different media at room temperature (30–33 °C) with shaking at 200 rpm for 5 days (**b**). The lowercase letters above the lines and vertical bars represent significant differences (*P* < 0.05) in the culture medium on each day. SEM micrograph (15,000×) of immobilized cells grown in different media for 1 day. Black arrows indicate biofilm secretion (**c**).

The biofilm-forming property is a dynamic process that facilitates cell adherence, resulting in stable cell immobilization. The results showed that the biofilm formation trends were increased in S103 from all tested media, 0.25xLB, 0.5%MK and 100%CW, after prolonged incubation (Fig. 2a). Surprisingly, the highest biofilm yield was obtained by S103 grown in 100%CW (Fig. 2a), in which the C/N ratio was higher than 0.5%MK and 0.25xLB (Supplementary Table S2). This corresponded to previous reports that biofilm formation was enhanced by an elevated C/N ratio^34^, which led to improved protection of bacteria and enhanced removal of pollutants^56,57^.

Furthermore, an appropriate medium for the immobilization of S103 on PUF was selected from 0.25xLB, 0.5%MK and 100%CW at RT and 200 rpm for 5 days. The results showed that the bacterial population of PUF-immobilized S103 in 100%CW surpassed the number of attached cells in 0.5%MK and 0.25xLB on the 2nd day, and the immobilized biomass maintained a steady growth state with 10^10^ CFU g^-1^ PUF until 5 days of immobilization (Fig. 2b). The increased PUF-immobilized S103 in 100%CW after prolonged incubation was related to efficient biofilm formation to facilitate strong surface attachment and colonization on the material surface (Fig. 2a, c and Supplementary Fig. S7). Moreover, this condition provided high numbers of attached cells on PUF in a shorter time of immobilization than long-term incubation in nutrient broth for 11 days to obtain *R. ruber* F92 as 10^9^ cells per cm^3^ PUF^24^. Thus, 100%CW was selected as the low-cost medium for immobilization with a high yield of biofilm in a short period for 3 days due to the time-saving immobilization required for cost-effectiveness of scale-up production.

### Scale-up production of biobooms in a modified FBB

**Figure 3:**
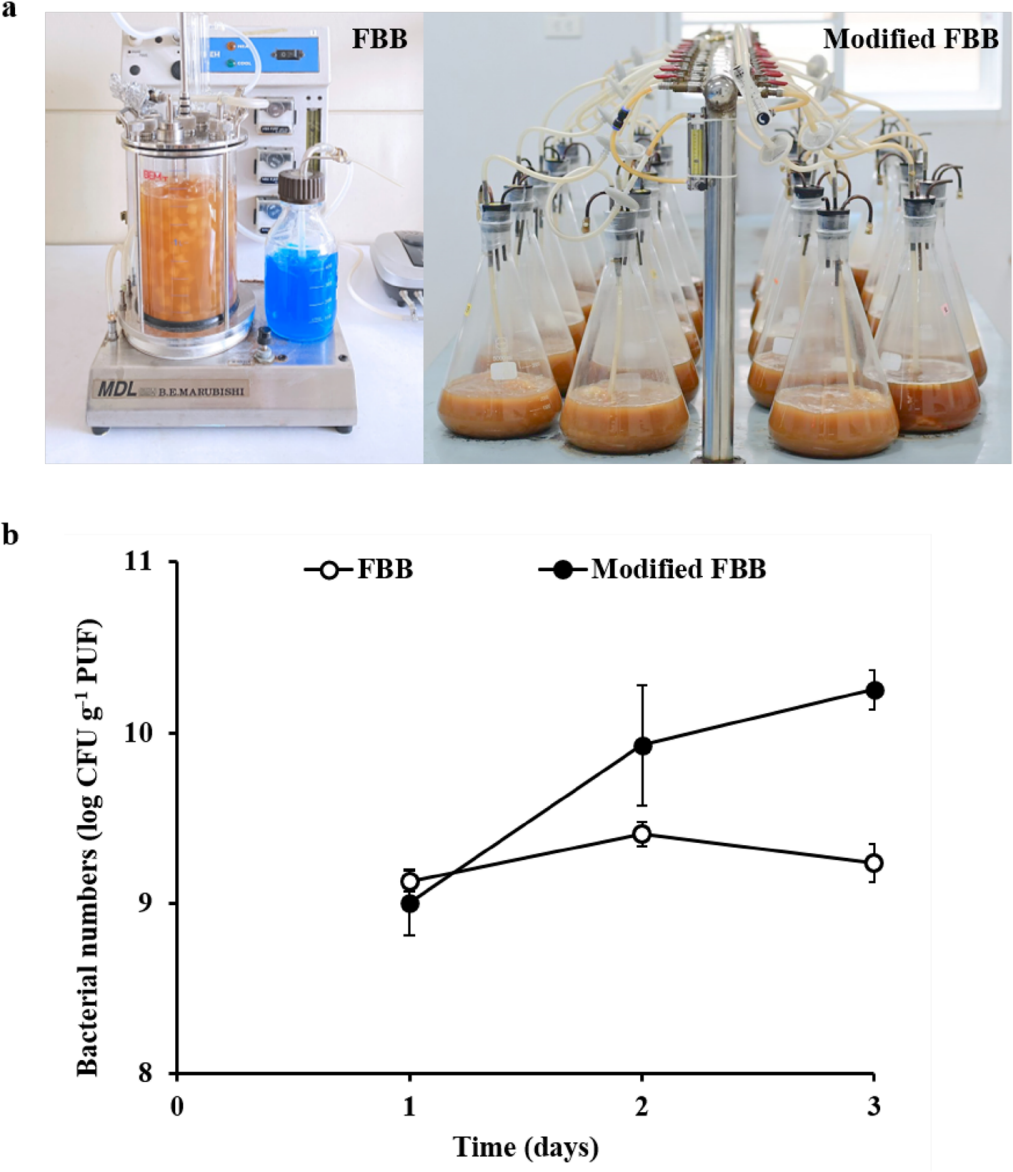
Image of FBR and the modified FBR for cell immobilization (**a**). Bacterial numbers of *R. ruber* S103 immobilized on PUF in 100%CW by a fluidized-bed bioreactor (FBB) and the modified FBB operated with filtered air at 1.50 and 2.86 L min^−1^, respectively, at room temperature for 3 days (**b**).

For commercial bioproducts, a simple system for the large-scale production of biobooms is needed. High amounts of immobilized S103 (>10^9^ CFU g^-1^ of PUF) were obtained from 2 L of FBB and 5 L of a modified FBB after 3 days of immobilization (Fig. 3). Under the same conditions, a modified FBB provided a high number of immobilized cells (1.85 ± 0.47 × 10^10^ CFU g^-1^ PUF) (Fig. 3b) as obtainable in a small flask. This corresponded to Kuyukina et al.^41^ reported that a high number of immobilized cells (approximately 10^7^ cells g^-1^ dry sawdust of cocultured *R. ruber* and *R. opacus*) were received from a modified FBB in a 500-mL Erlenmeyer flask after 7 days. Herein, 180 g (dry weight) of biobooms were simultaneously produced from 60 L of 100%CW in 20 sets of modified FBB, suggesting that the modified FBB is a powerful tool to produce immobilized cells because the system was easily constructed with a relatively low-cost operation and environmentally friendly technology. Regarding production costs, the price of 1 kg of dry biobooms from 350 L of alterative media or 0.25xLB medium was calculated (Table 3). The production cost is a significant reduction for 85%, if low-cost media are used for immobilization. Lo et al.^31^ showed that the use of a low-cost culture medium, corn steep liquor and molasses reduced 30% of the original media for biomass production of the plant growth-promoting *Rhodopseudomonas palustris* strain PS3, indicating that a simple bioreactor system is a reality to be useful for large-scale bioremediation.

**Table 3:** Comparison of costs for bioboom production.

Culture medium	Culture medium used (Liter)	Costs (US$) per liter	Costs (US$) per kg (dry boom)*
0.25xLB	350	0.975	341
0.5%MK	35	0.004	53
100%CW	315	0.167	

*30 THB is equivalent to 1 US$.

**Himedia™ Luria Bertani Broth (Miller).

### Efficiency of biobooms on crude oil removal on the flask scale

**Figure 4:**
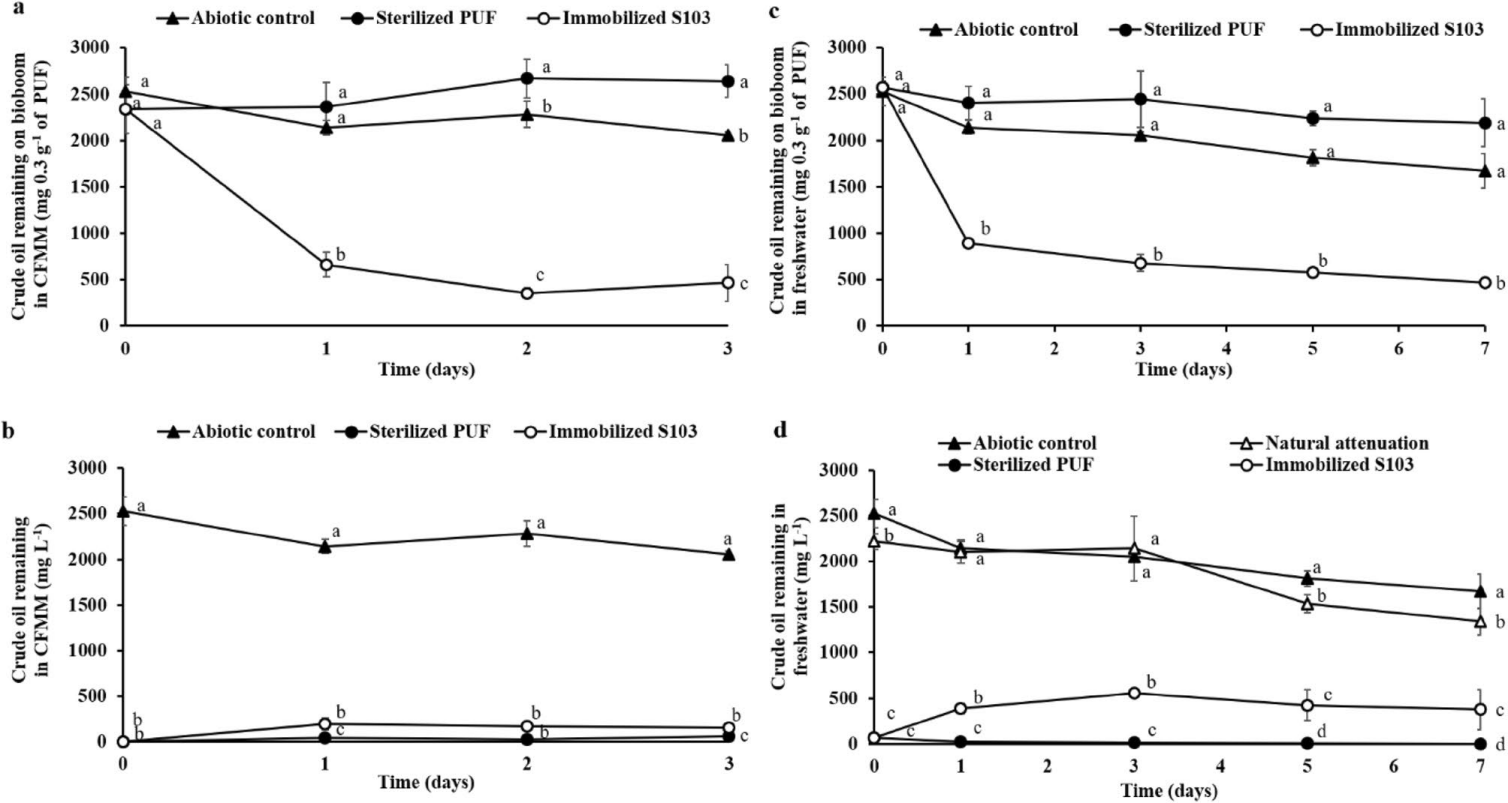
Removal efficiency of crude oil by *R. ruber* S103 on biobooms (**a** and **c**) and in liquid culture (**b** and **d**) and at room temperature with shaking at 200 rpm for 3 and 7 days. The removal was carried out in CFMM medium (**a** and **b**) and freshwater (**c** and **d**), which contained 2500 mg L^−1^ AL crude oil. The lowercase letters above the vertical bars represent significant differences in incubation time (*P* < 0.05).

The efficiency of crude oil removal by the biobooms was evaluated in CFMM and sterilized freshwater supplemented with 2500 mg L^−1^ crude oil (Fig. 4). The result showed that crude oil was rapidly removed on the first day of incubation, which was due to high adsorption capacity of PUF (10.8 ± 0.43 g g^−1^ carriers) as analyzed in the Supplementary Data (Text S3). When compared to nonbiological control of sterile PUF, the biobooms exhibited high crude oil biodegradation (77.1 ± 7.75%) in CFMM within 3 days (Fig. 4a). Crude oil was slightly evaporated by physical abiotic factors (Fig. 4b). Furthermore, crude oil removal by biobooms in freshwater without nutrient supplementation showed the same trends in the CFMM medium. Biobooms rapidly biodegraded the adsorbed crude oil on the first day, and biodegradation increased to 61.5 ± 8.11% in sterilized freshwater within 7 days (Fig. 4c), while only 19.6 ± 1.6% biodegradation occurred by an indigenous bacterium (natural attenuation) in the nonsterilized freshwater after 7 days (Fig. 4d), suggesting that bioaugmentation by biobooms effectively removed crude oil and simultaneously biodegraded the adsorbed crude oil even in a simulated environment. Additionally, the biobooms maintained more than 74% biodegradation ability of crude oil under various salinities (0 to 5% w/v sodium chloride) in CFMM after 3 days (Supplementary Fig. S8), suggesting its potential applicability for freshwater and marine environmental cleanup.

### Efficiency assessment of biobooms on crude oil removal in simulated environmental contamination

**Figure 5:**
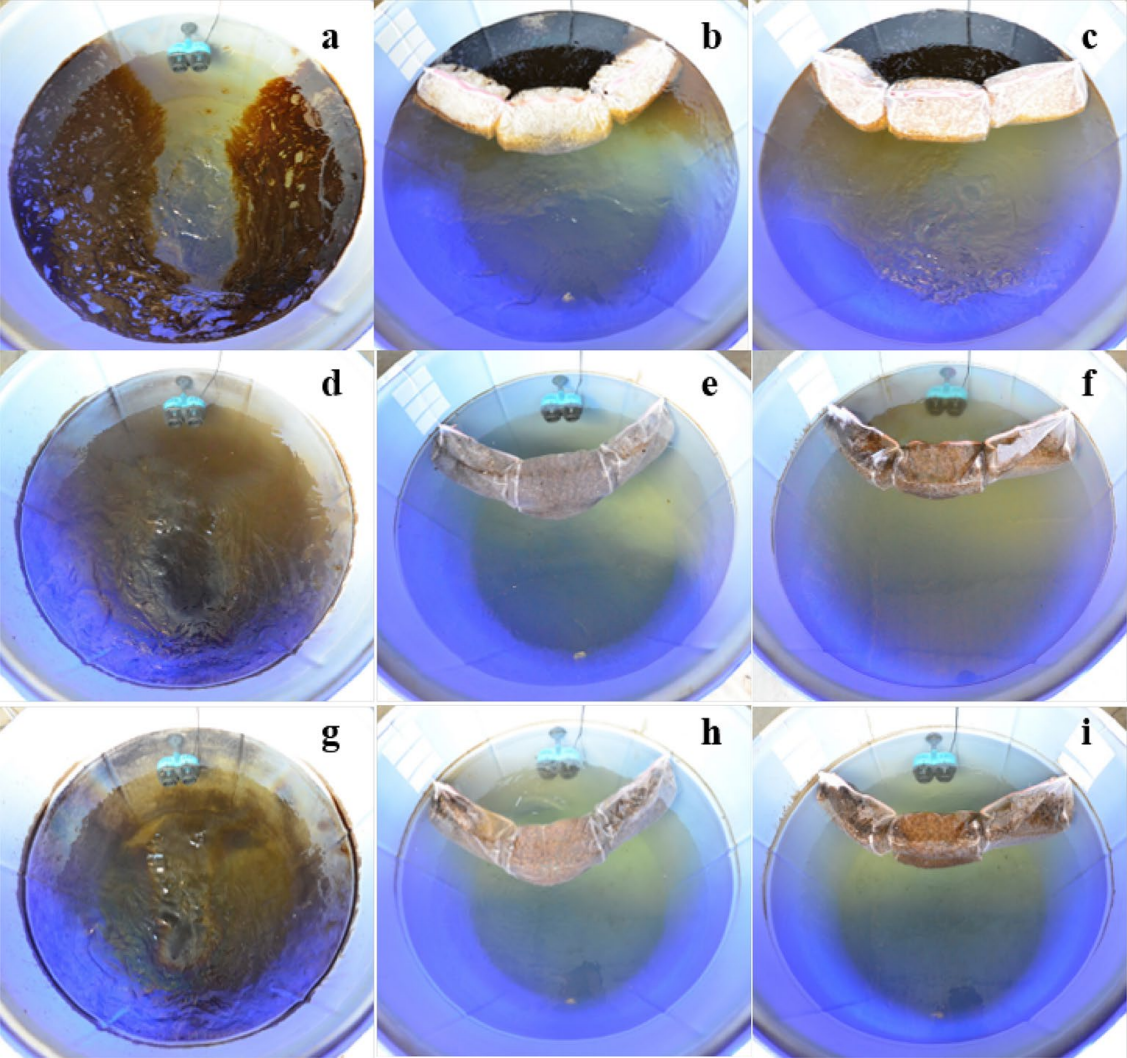
Visualization of crude oil removal by natural attenuation (**a**, **d** and **g**), control boom (**b**, **e** and **h**) and bioaugmentation with biobooms (**c**, **f** and **i**) in a simulated environmental mesocosm; 500-L freshwater added 72 g of AL crude oil, with artificial waves under natural conditions, wind, sunlight and room temperatures ranging from 30 to 35 °C for 0 (**a**–**c**), 3 (**d**–**f**) and 7 (**g**–**i**) days.

The experiment was performed in simulated freshwater mesocosms with artificial waves generated by a wave simulator under natural conditions, windiness, rain, sunlight and temperatures ranging from 30 to 35 °C. In natural attenuation, 72 g of crude oil was rapidly dispersed on the surface of freshwater on the first day of oil contamination compared to other tanks (Fig. 5a–c) since oil was detected from 4 sampling points of the surface water (Supplementary Table S3). Moreover, the oil was laterally dispersed by the simulated wave since oil trapped on the tank wall was observed after 3–7 days (Fig. 5d and g) but was not detected from the bottom of the tank (data not shown), indicating difficulty in controlling the contaminated area after oil spills in aquatic environments. In the remediated mesocosm, absorbent booms and biobooms could adsorb crude oil and narrow the contaminated area on the first day. Within only 3 days, crude oil was completely removed by adsorption on PUF from the freshwater (Fig. 5e, f, h and i) since oil was below the detection limit by GC-FID from the surface (Supplementary Table S3) as well as the middle and bottom of the tank (data not shown), suggesting that the use of the adsorbent PUF booms has the potential to mitigate the impact on living organisms in aquatic ecosystems. After 7 days, biobooms could biodegrade approximately 60% of the adsorbed oil when compared to boom control with indigenous bacteria and the survival of the immobilized cells was still high approximately 6.06 ± 2.21 × 10^7^ CFU g^-1^ of dry bioboom (Supplementary Table S4). This was because the oil bound to the sorbent acts as an energy and carbon source, indicating that biobooms could maintain high crude oil biodegradation efficiency even in simulated freshwater environments as obtainable from the flask scale (Fig. 4 and Supplementary Fig. S8). Regarding hydrocarbon degradation, many studies have been carried out over many days or even several months in microcosms^58–61^. Nevertheless, a few reports have evaluated the efficiency of the bioaugmentation of contaminated crude oil by immobilized cells in a mesocosm. Dellagnezze et al.^62^ showed that more than 90% degradation of total *n*-alkane within 10 days was obtained by chitosan-immobilized bacteria adhered to the walls of mesocosms, in which 72 mL of AL crude oil was dispersed in 3000 L seawater. To the best of our knowledge, the biobooms were a more effective bioproduct since it could maintain high biodegradation activity even when challenged directly and rapidly exposed to a high concentration of crude oil (72 g) in natural freshwater without any nutrient supplementation. Gertler et al.^7^ showed that a prototype oil boom including oil sorbents, slow-release fertilizers and biomass of the marine oil-degrading bacterium *Alcanivorax borkumensis* effectively removed heavy fuel oil (0.5% v/v) in a 500-L mesocosm after 56 days. This study supports the feasibility of using bioproducts as oil sorbent booms for the removal of various types of oils from water. Hence, the biobooms should be evaluated the practical bioremediation of crude oil contaminated in realistic open water ecosystems that will provide a beneficial impact after an oil spill.

### Evaluation of storage stability of bioboom product

**Figure 6:**
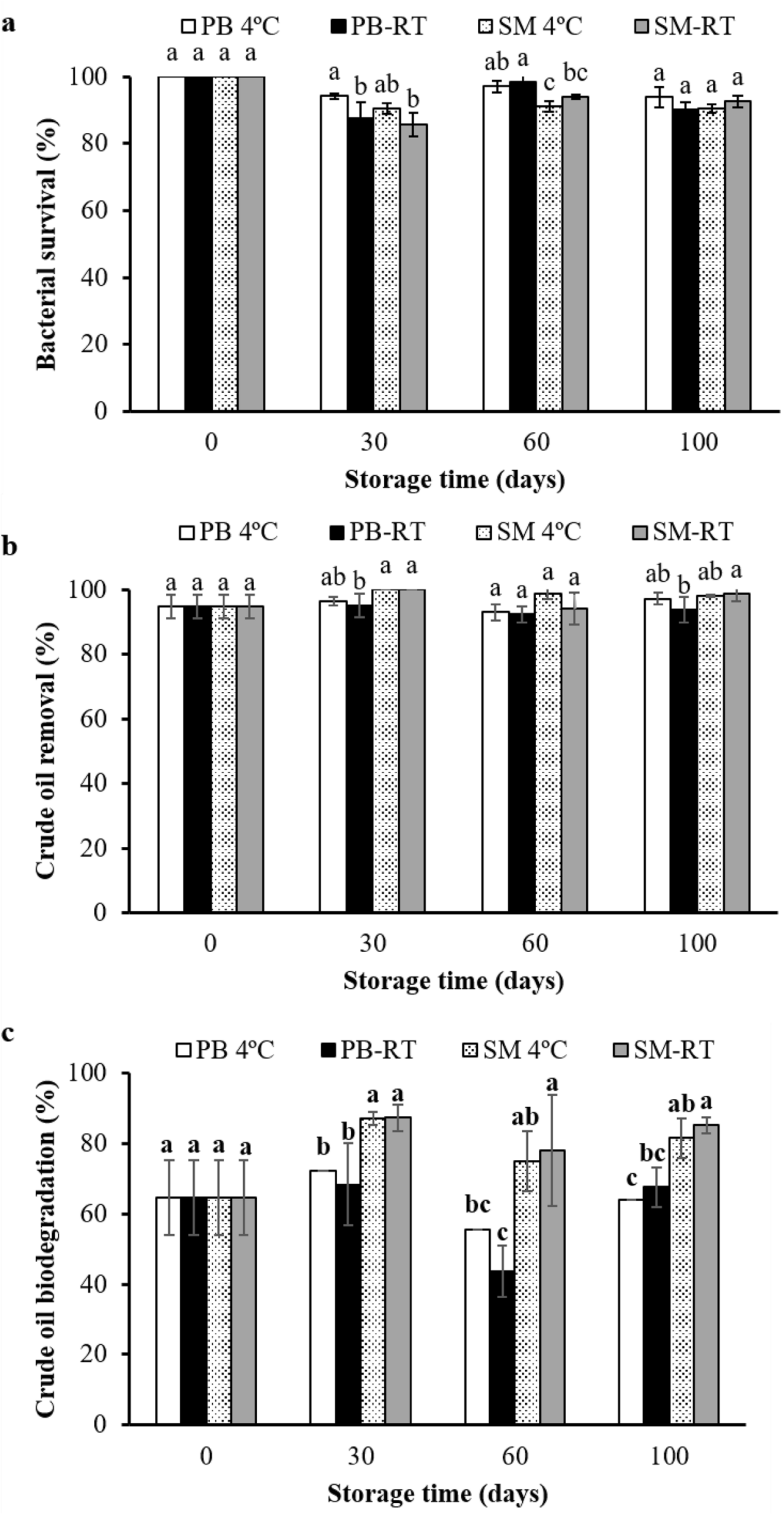
Bacterial survival (%) of immobilized *R. ruber* S103 after storage for 30, 60 and 100 days in phosphate buffer (pH 6.5) and 10% (w/v) skimmed milk at 4 and room temperature, 30–33 °C (**a**). Crude oil removal from the medium (**b**) and biodegradation of the adsorbed oil by immobilized S103 after storage were carried out in CFMM medium supplemented with 2500 mg L^−1^ AL crude oil at room temperature with 200 rpm for 3 days. The lowercase letters above the vertical bars represent significant differences in storage conditions (*P* < 0.05).

The shelf life is a major considerable factor for commercial bioproducts. After 30, 60 and 100 days of storage, the cell survival and crude oil-degrading ability of the immobilized S103 on biobooms were estimated. The cell viability of the immobilized S103 was more than 85% (> 2.8 ± 1.6 × 10^8^ CFU g^−1^ PUF) after storage in both PB and SM at 4 °C and RT (Fig. 6a). After 100 days of storage under all conditions, the biobooms maintained above 92% crude oil removal efficiency by adsorption on PUF (Fig. 6b). The biobooms stored in SM at both temperatures biodegraded the adsorbed crude oil better than freshly immobilized cells (Fig. 6c). Similarly, in a recent study, the starvation state of cells was obtained after prolonged storage, resulting in faster hydrocarbon biodegradation than with fresh cells^47^. Furthermore, the biobooms preserved in SM at RT for 100 days was still active and provided the highest crude oil biodegradation (85.2 ± 2.3%) in CFMM at 3 days (Fig. 6c). According to Cody et al.^63^, over 90% of strains frozen in 10% skimmed milk were recovered. Suo et al.^64^ showed that 20 or 30% (w/w) skimmed milk demonstrated significant improvements in cell survival (remaining viability > 10^8^ CFU mL^−1^) and growth capability of lactic acid bacteria after heat treatment at 65 °C for 10 min. Thus, the skimmed milk is an effective buffer for enhanced viability of cells after preservation over a wide temperature range. Therefore, the storage of biobooms at RT has advantages with regard to their simple preparation and cost-effectiveness.

## Conclusions

In this study, nonpathogenic *R. ruber* S103 was proven to be a promising strain to develop an effective bioproduct for the bioremediation of crude oil. This strain obviously exhibited efficient biodegradation of high concentrations of crude oil. For economically viable bioproducts, alternative low-cost media, including molasses (formulated with adequate inorganic phosphate buffer) and mature coconut water, were successfully used for enhanced biomass of S103 during inoculum preparation and immobilization on PUF, respectively. The use of low-cost media for the cultivation of S103 had positive physiological responses to resist toxicity and increase the biodegradation of crude oil in freshwater and even in up to 5% (w/v) NaCl. Moreover, the immobilization of S103 on PUF as biobooms was perfectly scaled up to obtain high amounts of PUF-attached cells in a modified fluidized-bed bioreactor. The production of biobooms using inexpensive organic substrates coupled with a simple bioreactor system could significantly reduce 85% of production costs. Moreover, bioaugmentation employing biobooms in a simulated freshwater mesocosm showed that the polluted crude oil was completely adsorbed, resulting in mitigation of the impacts on living aquatic organisms, and the adsorbed oil was simultaneously biodegraded, leading to a reduction in secondary contamination from elimination of contaminated materials. Furthermore, the biobooms still maintained biodegradation after prolonged storage under simple operation. In summary, the biobooms, the combination of effective bacterial cells plus low-cost production, is a very promising tool that deserves further evaluation for sustainable bioremediation of crude oil polluted aquatic environments.

## Supplementary Information

Supplementary Information.

## Data Availability

All data generated or analyzed during this study are included in this published article and its supplementary information files.
