# Supplementary material for: Physiological changes in Rhodococcus ruber S103 immobilized on biobooms using low-cost media enhance stress tolerance and crude oil-degrading activity
Source: Sci Rep. 2022 Jun 21;12:10474. doi: 10.1038/s41598-022-14488-0 (PMC9213463; doi:10.1038/s41598-022-14488-0)
Supplement: Supplementary file 1 — Supplementary Information. [file 41598_2022_14488_MOESM1_ESM.pdf]

Supplementary Data

Title: Physiological changes in *Rhodococcus ruber* S103 immobilized on biobooms using low-cost media enhance stress tolerance and crude oil-degrading activity

Authors: Kallayanee Naloka[1,2], Jirakit Jaroonrunganan[1,2], Naphatsakorn Woratecha[1,2], Nichakorn Khondee[3], Hideaki Nojiri[4,5] and Onruthai Pinyakong[1,2]*

[1]Center of Excellence in Microbial Technology for Marine Pollution Treatment (MiTMaPT), Department of Microbiology, Faculty of Science, Chulalongkorn University, Bangkok 10330, Thailand

[2]Research Program on Remediation Technologies for Petroleum Contamination, Center of Excellence on Hazardous Substance Management (HSM), Chulalongkorn University, Bangkok 10330, Thailand

[3]Department of Natural Resources and Environment, Faculty of Agriculture, Natural Resources and Environment, Naresuan University, Phitsanulok 65000, Thailand

[4]Agro-Biotechnology Research Center, Graduate School of Agricultural and Life Sciences, The University of Tokyo, Tokyo, 113-8657, Japan

[5]Collaborative Research Institute for Innovative Microbiology, The University of Tokyo, Bunkyo-ku, Tokyo, 113-8657, Japan

***Corresponding author:** Onruthai Pinyakong

Tel.: +662-218-5070; Fax: +662-252-7576

E-mail: onruthai.p@chula.ac.th, onruthai@gmail.com; ORCID: 0000-0003-0014-1690

Text S1: Arabian light (AL) crude oil source and properties

Arabian light (AL) crude oil was kindly provided by the Thai Oil Public Company Limited (Thailand). The viscosity, density and API gravity were 13 cP, 0.86 g cm^{-3} and 31° API, respectively[1]. The composition was a mixture of 40% saturated hydrocarbons, 26% aromatic hydrocarbons, 24% resins and 10% asphaltenes, which was analyzed by thin-layer chromatography with the flame ionization method (Iatron Labs, Tokyo, Japan) according to Nopcharoenkul, et al.[2].

Text S2: Selection of low-cost media

The growth characteristics of *Rhodococcus ruber* S103 were preliminarily determined in different concentrations of molasses and mature coconut water (CW) using 96-well plates at room temperature (30-33 ºC). The inoculum (20 µL) was added to 180 µL of the tested media to obtain an initial cell concentration of 10^7 CFU mL^{-1}. After incubation, the growth of S103 was measured for optical density at 540 nm using a multimode plate reader (PerkinElmer, Finland). S103 grew well in 100%CW, 0.25xLB and 0.5-1.0%M, respectively (Fig. S2). The lowest growth of S103 was obtained from 0.5-1.0%M since this condition might be an unfavorable pH condition. S103 preferred the optimum pH for growth as neutral to mildly alkaline conditions (Fig. S3). This strain could not grow in 100%CW (pH ~5) without pH adjustment (data not shown). The pH of the sterilized CW could be maintained after adjustment with NaOH to obtain pH 7.0, while the pH of molasses dissolved in distilled water still changed after sterilization (121 ºC, 15 min). Saejung and Puensungnern[3] observed a drastic pH drop from 6.8 (initial pH) to 3.4 in the culture broth when molasses concentrations ranged from 2 to 10 g L^{-1}, at which buffering action was needed. Thus, low-cost inorganic phosphate salts were added to maintain a pH of 0.5%M. The growth of S103 was promoted in 0.5%M added 0.26% (w/v) K_2HPO_4, named as 0.5%MK, when compared to other phosphate salts, and the pH was

adjusted with 1 M NaOH before and after sterilization (Fig. S4). It is important to note that the use of only raw materials should be considered pH control, especially to scale up biomass production. In this study, 100%CW and 0.5%MK were selected for further study prior to being used as alternative media.

Text S3: Crude oil adsorption capacity

The oil adsorption capacity was determined using a slightly modified method from Songsaeng, et al.[4] . Briefly, 2 g crude oil was added into 30 mL of freshwater. The PUF cubes (1x1x1 cm^3) were weighed before their immersion into the oil-freshwater system at room temperature. After 20 min, the PUF was removed from the system. The excess oil on the PUF surface was removed, and the PUF were weighed. All experiments were performed in triplicate. The adsorption capacity was calculated as follows: adsorption capacity ($g\ g^{-1}$) = $(W_1-W_0)/W_0$, where W_0 and W_1 represent the weight of before and after adsorption of PUF, respectively.

References

1 Hollebone, B. P. in *Handbook of Oil Spill Science and Technology* (ed Mervin Fingas) 575-681 (John Wiley & Sons, Inc., 2015).

2 Nopcharoenkul, W., Netsakulnee, P. & Pinyakong, O. Diesel oil removal by immobilized *Pseudoxanthomonas* sp. RN402. *Biodegradation* **24**, 387-397, https://doi.org/10.1007/s10532-012-9596-z (2013).

3 Saejung, C. & Puensungnern, L. Evaluation of molasses-based medium as a low cost medium for carotenoids and fatty acid production by photosynthetic bacteria. *Waste Biomass Valor.* **11**, 143-152, https://doi.org/10.1007/s12649-018-0379-6 (2020).

4 Songsaeng, S., Thamyongkit, P. & Poompradub, S. Natural rubber/reduced-graphene oxide composite materials: Morphological and oil adsorption properties for treatment of oil spills. *J. Adv. Res.* **20**, 79-89, https://doi.org/10.1016/j.jare.2019.05.007 (2019).

Table S1 Physicochemical properties of freshwater collected from the Chao Phraya River and the Tha Khoei Canal.

**Parameter**	Chao Phraya River	Tha Khoei Canal
pH	7.60	7.20
Salinity (g L^{-1})	0.10	0.00
Chemical oxygen demand or COD (mg L^{-1})	17.98	23.9
Biological oxygen demand or BOD (mg L^{-1})	<1.0	0.88
Total nitrogen (mg L^{-1})	0.42	0.63
Total phosphate (mg L^{-1})	0.11	0.12
Potassium (mg L^{-1})	5.35	4.82
Iron (mg L^{-1})	0.11	1.17
Mercury (mg L^{-1})	<0.001	<0.001
Magnesium (mg L^{-1})	Not determined	11.0
Calcium (mg L^{-1})	Not determined	4.42

Table S2 Nutrient compositions in low-cost media.

Parameter	Molasses	CW	Unit
Protein (N x 6.25)	6.61	0.12	$g\ 100\ g^{-1}$
Total sugar	40.18	2.02	
Fructose	9.17	nd	
Glucose	3.78	2.02	
Sucrose	27.23	nd	
Maltose	nd	nd	
Lactose	nd	nd	
Ash	10.09	0.50	
Sodium (Na)	97.39	31.59	$mg\ 100\ g^{-1}$
Calcium (Ca)	1,050.28	17.27	
Iron (Fe)	9.43	0.42	
Magnesium (Mg)	512.39	8.42	
Potassium (K)	2,656.84	170.02	
Total organic acid	7.11	4.09	$g\ L^{-1}$
L-Ascorbic acid	1.19	nd	
Lactic acid	3.98	1.75	
Acetic acid	0.79	0.90	
Citric acid	nd	nd	
Succinic acid	nd	1.44	
Brix	84.80	4.13	^{o}Brix
pH	5.5	5.3-5.5	-

nd = not detected (Limit of detection: 0.10 g 100 g^{-1} and 0.1 mg L^{-1})

The nutrient compositions of both alternative substrates were analyzed using high-performance liquid chromatography by the Food Research and Testing Laboratory, Department of Food Technology, Faculty of Science, Chulalongkorn University.

Table S3 Remaining crude oil analyzed by GC-FID from 4 sampling points of the surface freshwater in natural attenuation, control and bioaugmentation (bioboom).

Time (days)	Natural attenuation					Control					Bioaugmentation (Bioboom)				
	**S1**	**S2**	**S3**	**S4**	***Remaining (mg L^{-1})**	**S1**	**S2**	**S3**	**S4**	***Remaining (mg L^{-1})**	**S1**	**S2**	**S3**	**S4**	***Remaining (mg L^{-1})**
0	316	781	1884	2662	5643	11459	16708	0	0	28168	9982	13715	0	0	23698
3	0	0	0	0	0	0	0	0	0	0	0	0	0	0	0
7	0	0	0	0	0	0	0	0	0	0	0	0	0	0	0

Remark: S1-S4, sampling points from surface water, *Sum of remaining crude oil detected from the surface area.

Table S4 Remaining crude oil analyzed by GC-FID from PUF collected in triplicate from control and bioaugmentation (bioboom) and survival of the bioboom-immobilized S103.

**Time (days)**	**Control (Boom)**					**Bioaugmentation (Bioboom)**					**Bacterial number**
	**1**	**2**	**3**	***Remaining ($mg\ g^{-1}$ PUF)**	**SD**	**1**	**2**	**3**	***Remaining ($mg\ g^{-1}$ PUF)**	**SD**	**CFU g^{-1} PUF**
0	69.7	183.9	35.8	**96.5**	**77.6**	143.0	64.5	52.6	**86.7**	**49.1**	**$8.21 \pm 1.18 x 10^9$**
3	314.7	217.5	197.3	**243.2**	**62.8**	348.8	73.7	143.8	**188.8**	**143.0**	**$1.26 \pm 0.27 x 10^8$**
7	105.9	191.9	50.8	**116.2**	**71.1**	91.8	14.2	48.3	**51.4**	**38.9**	**$6.05 \pm 2.21 x 10^7$**

1, 2 and 3: the code of each boom and bioboom, *Average of the remaining crude oil detected from the boom and bioboom in triplicate.

a

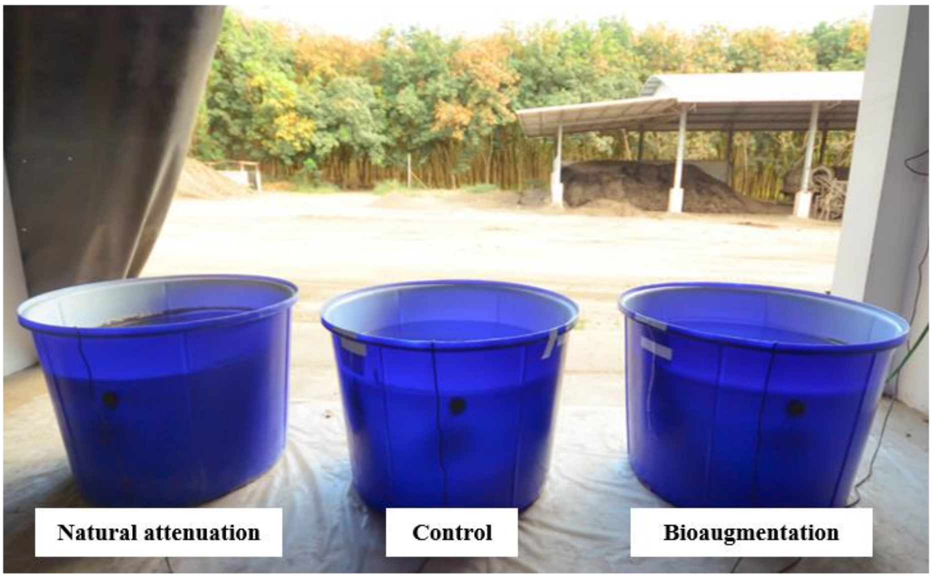

b

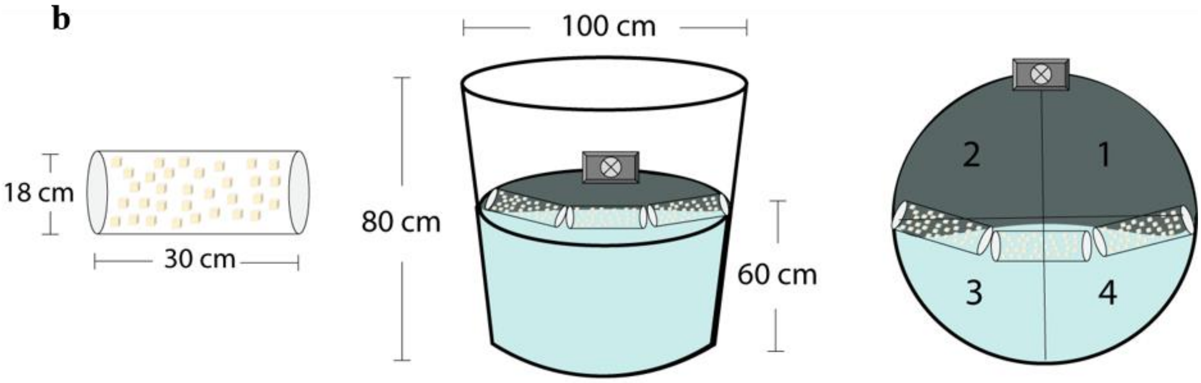

Fig. S1 Actual experimental tanks of the simulated freshwater environments, natural attenuation, control and bioaugmentation under natural conditions used in this study (a). Schematic representation of crude oil remediation in mesocosm tanks (control and bioaugmentation) (b).

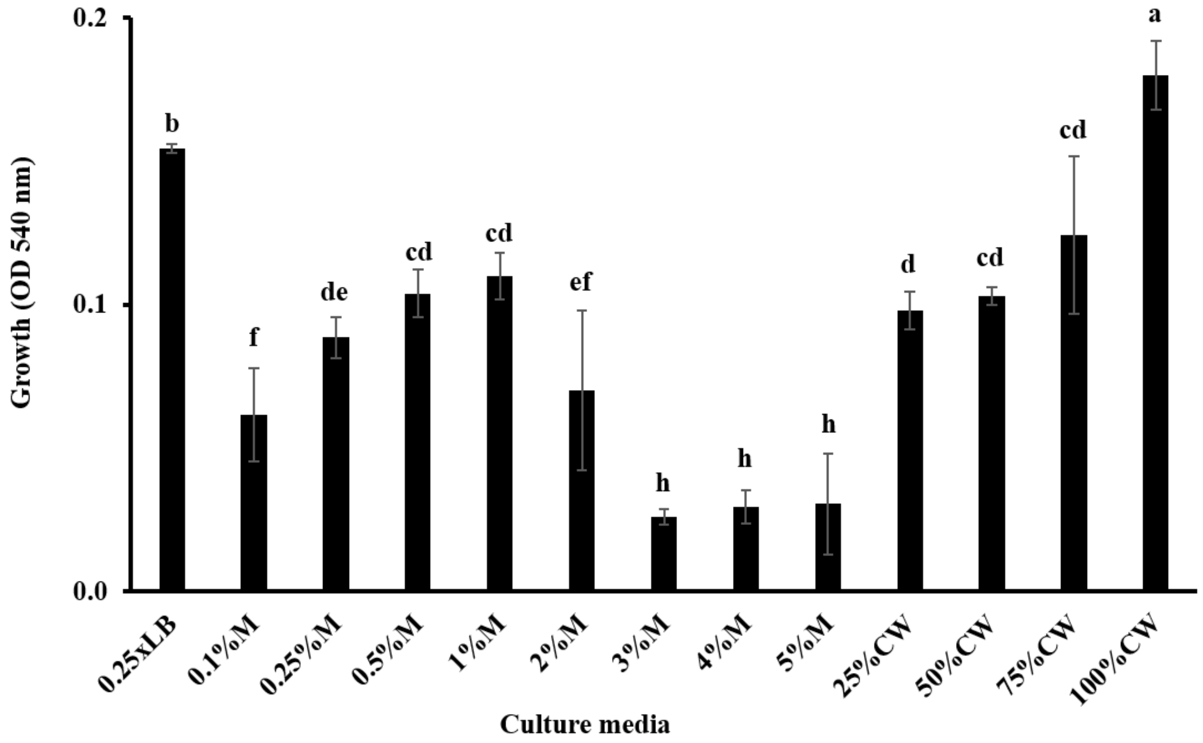

Fig. S2 Growth characteristics of *R. ruber* S103 in sterilized molasses (pH ~5.8) and mature coconut water (pH ~7). Preculture of S103 was prepared in the same medium. Growth was performed in 96-well plates at room temperature for 24 h and determined by measuring the optical density at 540 nm. The lowercase letters above the vertical bars represent significant differences in the culture media ($P < 0.05$).

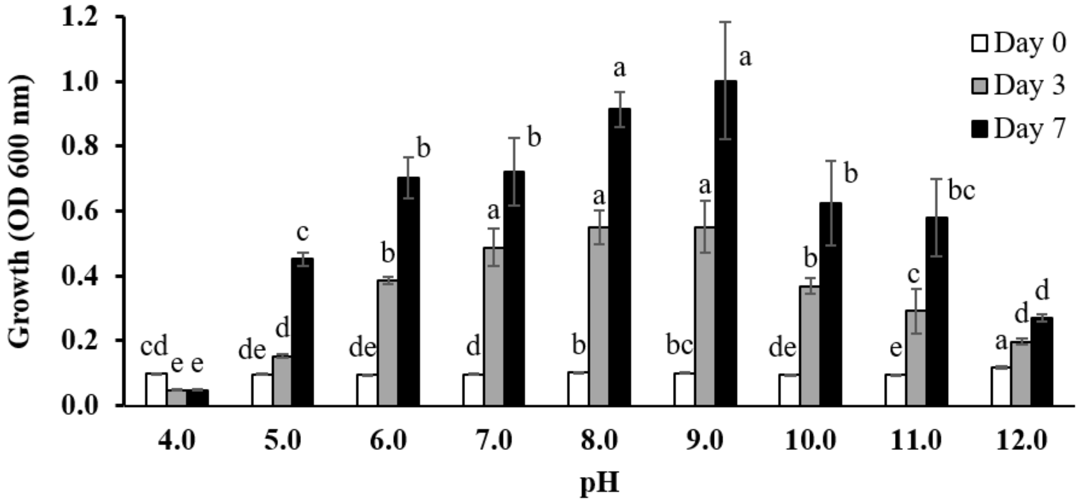

Fig. S3 Growth characteristics of *R. ruber* S103 determined by the most probable number (MPN) in LB medium (pH 4-12) at room temperature for 7 days. The different letters on the same day represent significant differences in pH ($P < 0.05$).

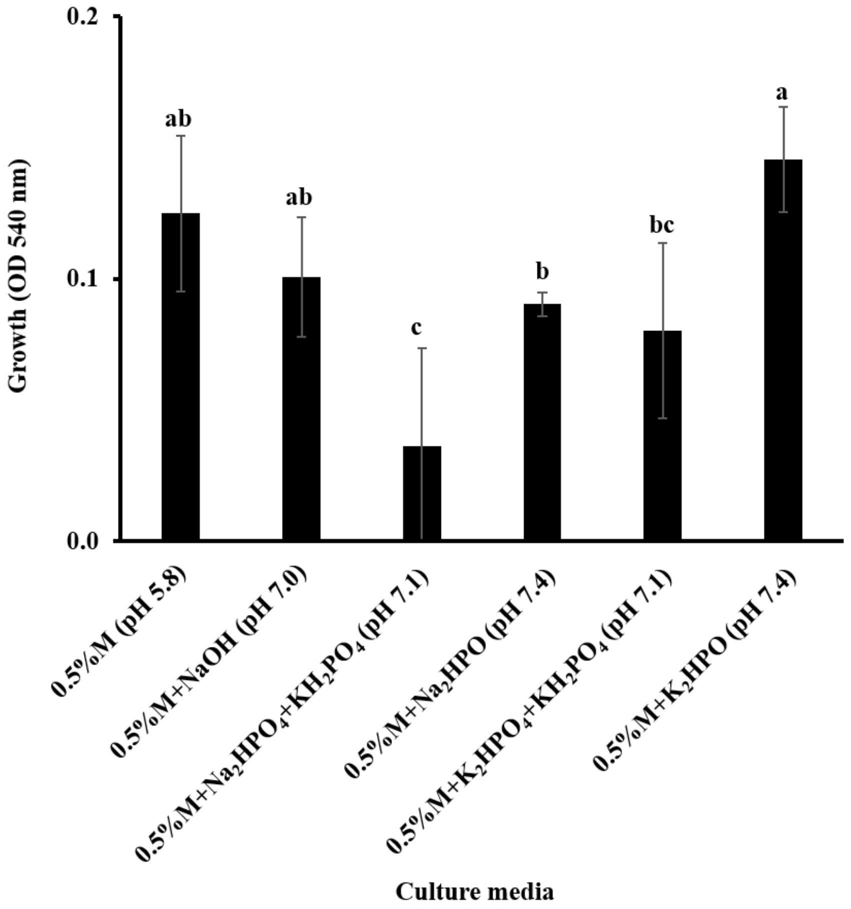

Fig. S4 Growth characteristics of *R. ruber* S103 in sterilized molasses (pH ~5.8) and sterilized molasses with inorganic phosphate salts (0.56% $Na_2HPO_4 \cdot 12H_2O$, 0.05% KH_2PO_4 and 0.26% K_2HPO_4). Preculture of S103 was prepared in the same medium. Growth was performed in 96-well plates at room temperature for 24 h and determined by measuring the optical density at 540 nm. The lowercase letters above the vertical bars represent significant differences in the culture media ($P < 0.05$).

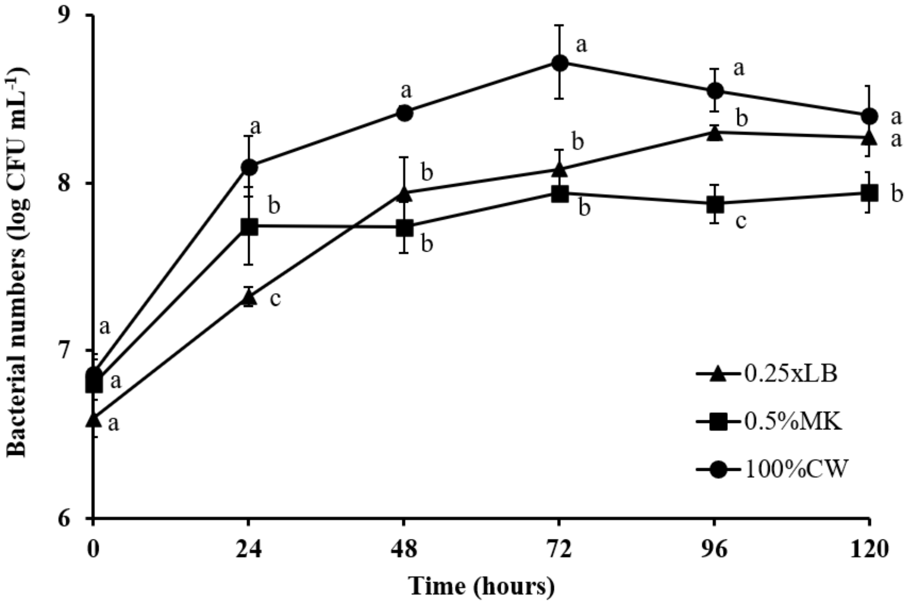

Fig. S5 A typical time course of growth of *R. ruber* S103 in 0.25xLB and low-cost media, 0.5%MK and 100%CW. Growth conditions were performed in 250-mL Erlenmeyer flasks containing 120 mL of medium at room temperature and 200 rpm for 120 h. The different letters on the same day represent significant differences at $P < 0.05$.

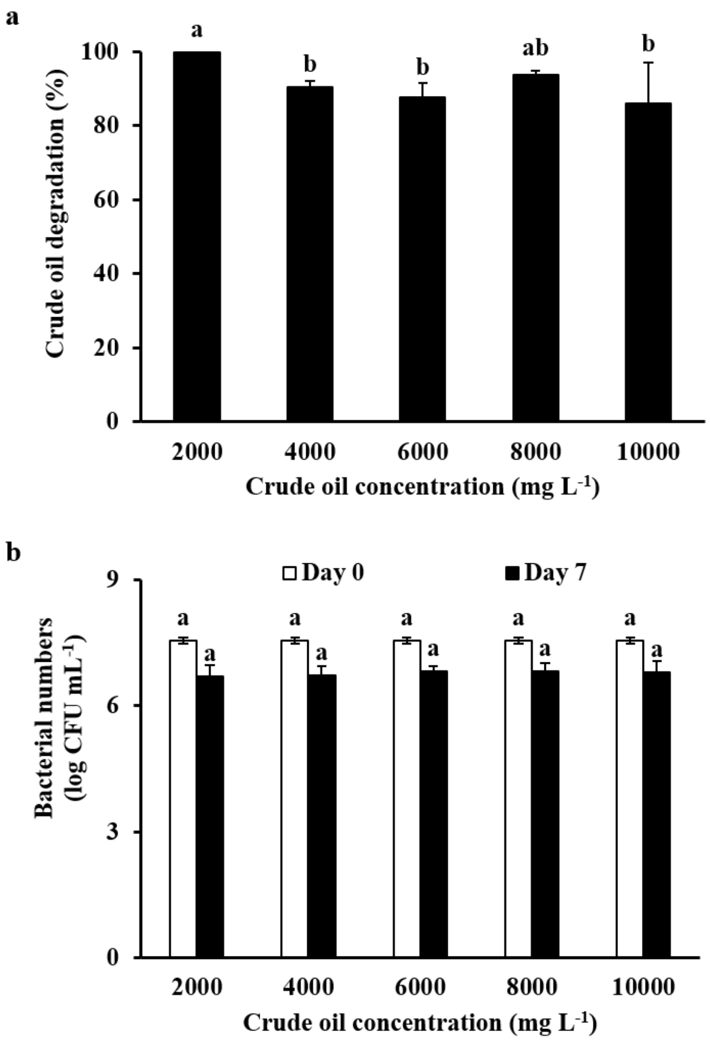

Fig. S6 Biodegradation efficiency of crude oil (a) and bacterial numbers (b) of *R. ruber* S103 in CFMM medium containing various concentrations of AL crude oil at room temperature and 200 rpm for 7 days. A preculture was grown in 0.25xLB. The lowercase letters above the vertical bars represent significant differences in the crude oil concentration ($P < 0.05$).

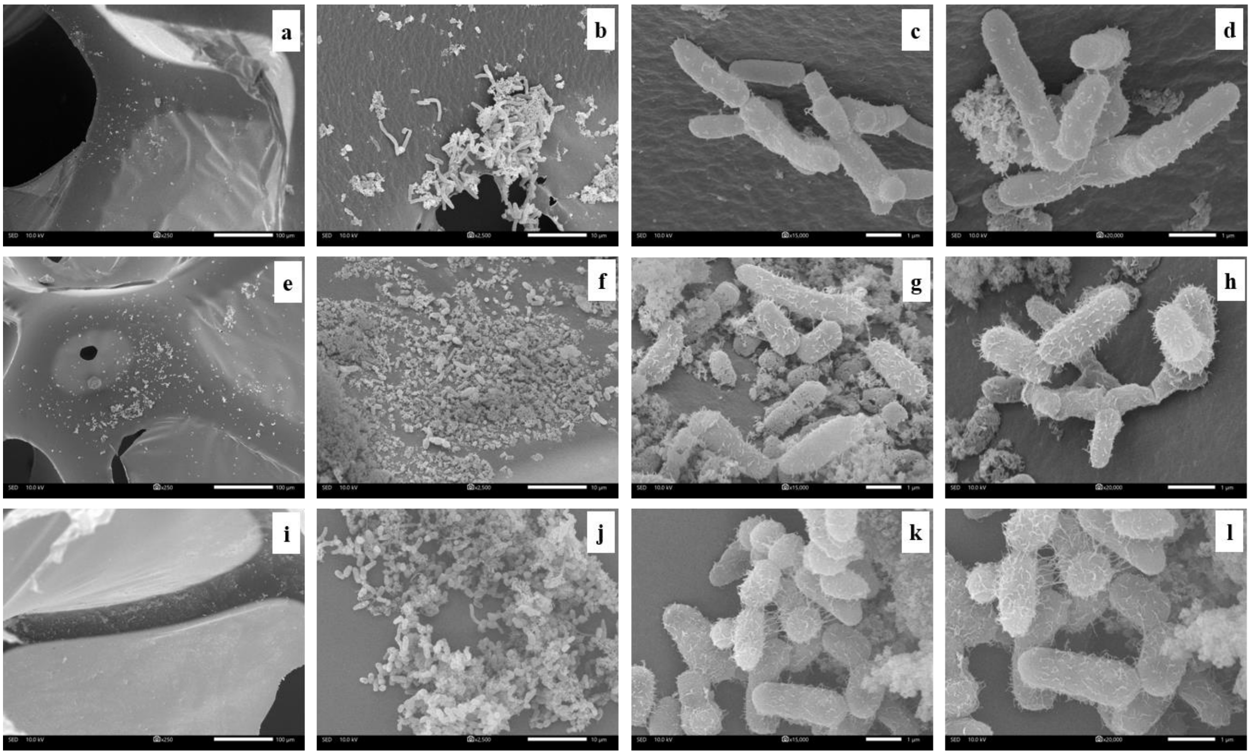

Fig. S7 A scanning electron micrograph at different magnifications, 250x (a, e and i), 2,500x (b, f and j), 15,000x (c, g and k) and 20,000x (d, h and l) of S103 immobilized on PUF in 100%CW at room temperature and 200 rpm for 1 (a-d), 3 (e-h) and 5 (i-l) days.

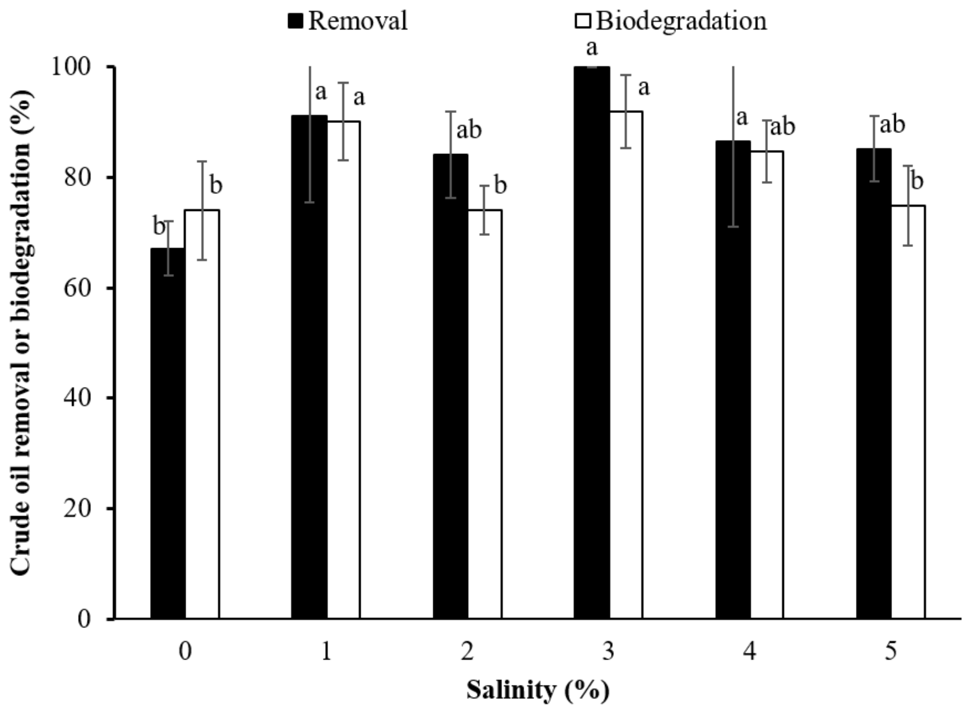

Fig. S8 Removal and biodegradation efficiency of crude oil by biobooms in CFMM with 0-5% (w/v) NaCl and 2,500 mg L^{-1} AL crude oil at room temperature with shaking at 200 rpm for 3 days. The lowercase letters above the vertical bars represent significant differences in NaCl concentrations ($P < 0.05$).
